# Factors associated with the utilization of long-acting reversible contraceptives among family planning clients at the Pameungpeuk Rural Hospital, Indonesia

**DOI:** 10.12688/f1000research.15755.2

**Published:** 2019-09-17

**Authors:** Achmad Kemal Harzif, Ana Mariana, Devi Marischa Malik, Melisa Silvia, Bara Tracy Lovita

**Affiliations:** 1 Division of Immuno-Endocrinology and Fertility, Department of Obstetrics and Gynecology, Faculty of Medicine Universitas Indonesia, Dr. Cipto Mangunkusumo Hospital, Jakarta, DKI Jakarta Province, Indonesia; 2 Indonesian Reproductive Medicine Research and Training Center (INA- REPROMED), Faculty of Medicine, Universitas Indonesia, Dr. Cipto Mangunkusumo Hospital, Jakarta, DKI Jakarta Province, Indonesia; 3 Department of Obstetrics and Gynecology, Persahabatan Public Hospital, Jakarta, DKI Jakarta Province, Indonesia

**Keywords:** Family Planning, Long-acting reversible contraceptives (LARCs), Pameungpeuk Rural Hospital

## Abstract

**Background**: Uncontrolled population development can prompt an assortment of populace issues and can be one of the reasons for increasing maternal death rates. The utilization of contraceptives in Indonesia was progressively dominated by injectable contraceptives and pill contraceptives in 2015 (52.21% and 24.36%, respectively). However, the rate of termination of the use of short-acting contraceptives by family planning clients was higher than other methods, therefore the use of short-acting contraceptives is less efficient than long acting reversible contraceptives (LARCs)  for longer term spacing because it is easy to skip a treatment for economic or other reasons, which can result in unintended pregnancy. Therefore, the National Family Planning Program in Indonesia is encouraging the use of LARCs to control population growth. Pameungpeuk is a region which has the second largest population, with the highest total fertility rate in South-West Java. The proportion of active users of LARCs in Pameungpeuk is very low (10.66%). This study aimed to analyze factors associated with the utilization of LARCs among family planning clients at the Pameungpeuk Rural Hospital.

**Methods**: This study design was cross-sectional with systematic random sampling. The sample group in this study was 84 family planning clients. We performed statistical analyses using chi-square test.

**Results**: We found significant associations between the age of women (p=0.024), the cost of contraception (p=0.022), knowledge (p=0.042), beliefs (p=0.002), skill of health workers (p=0.008) and support from health workers (p=0.014). However, education (p=0.212), family income (p=0.087), attitude (p=0.593), exposure to information on LARCs (p=0.378), support from partners (p=0.094), support from friends (p=0.414) and the support of community leaders (p=0.367) had no significant association with the utilization of LARCs.

**Conclusions**: These findings highlight a critical need for improved education among family planning clients at the Pameungpeuk rural hospital regarding the use of LARCs for both medical and elective reasons.

## Introduction

According to the World Population Data Sheet (2013), Indonesia is the fourth most populous country in the world, with a population estimate of 249 million. Among Association of South East Asian Nations (ASEAN) countries, Indonesia has both the largest area and largest population. In terms of birth rate, Indonesia is far above the 9 other member countries with a total fertility rate (TFR) of 2.6, while the average TFR of ASEAN countries is 2.4 ^1^.

Increased and uncontrolled population growth can be one of the reasons behind increasing maternal death rates. Demographic and Health Survey (DHS) information from 2007 set up the Indonesian maternal death rates as 228 deaths for every 100,000 live births ^2^. It is estimated that the global maternal death rates will be 195 deaths per 100,000 live births, while the proportion of maternal death in females reproductive age (PMDF) estimated will be 264–285 deaths per 100,000 live births in 2015 ^3^. The maternal death rate in Indonesia is high, and was recently estimated as 126 deaths per 100,000 live births by the World Bank. As an effort to improve maternal health and also achieve the fifth Millennium Development Goal, it required a reduction in maternal death by 9.5% every year from 2005 to 2015, but in reality this goal was not achieved between 2005–2008 ^3, 4^.

Low contraceptive use is associated with high maternal morbidity and mortality rates, unwanted pregnancies, short birth interval and higher risk of obstetric and newborn complications ^5^. One of the Indonesian government’s efforts to reduce population growth and its associated problems, including high mortality rates, are family planning programs promoting contraceptive use, which educate patients and clients regarding family planning and contraception itself ^4^. Based on data from the WHO, compared to other ASEAN countries, the use of contraceptives in Indonesia (61% of the population) already exceeds the ASEAN average (58.1%). However, it is still lower than that in Vietnam (78%), Cambodia (79%) and Thailand (80%) ^6^. Based on data from the DHS (2012), the use of contraception in Indonesia is dominated by injectable contraceptives (32%) and the pill (14%).

In 2010–2014, the National Family Planning Program in Indonesia directed more efforts towards promoting the use of long-acting reversible contraceptives (LARCs). LARCs are methods of contraception that are known to be highly effective, as they can provide protection from the risk of pregnancy for a period of up to 10 years, depending on the type used ^6, 7^.

The advantages of using long-term contraceptive methods are their convenience, comparatively low cost over time and predominant effectiveness in preventing pregnancy compared to short-acting methods. LARCs include implants, intra uterine devices (IUDs), male operation methods (MOPs) and female operation methods (MOWs) ^8^. The use of LARCs is proposed as an important strategy to reverse undesirable maternal health consequences in developing countries ^9, 10^. Use of injectable has been predominantly high while IUD utilize has rapidly low in developing countries ^11, 12^. Based on data from the DHS (2012), the proportion of LARC users in Indonesia declined between 1994 and 2012. In 2012, the total number of LARC users was 10.6%, while the national target was 27.5% ^6^.

Based on the 2015 results of the State Ministry for Population and National Family Planning Coordinating Board (BKKBN), contraceptive use in Indonesia is increasingly dominated by short-acting contraceptives such as injectables (52.21%) and the pill (24.36%). The total number of LARC users in 2015 was (18.17%), while the majority used short-acting contraceptives (81.83%) ^13^. In BKKBN 2017 shown that the realization of modern contraceptive prevalence rate such as use of female sterilization (MOW), male sterilization (MOP), pill, IUD, injection, implant KB (Implant) and condoms on 2017 is 57.6% of the target of 60.9% or achievement of 94.58%. Furthermore, the realization of unmet need which is defined as the percentage of married women who do not want to have more children but do not use contraception in 2017 is 17.5%, so that the achievement is 58.63%. Moreover, the percentage of active contraceptive participants using LARCs was 21.5% of the target of 21.7% or achievement of 99.07%. Additionally, the realization of contraceptive discontinuation rate in 2017 is 22.3% of the target of 25.3% or achievement of 88.14% ^14^. Pameungpeuk is a region with the second largest population, and has the highest TFR in South-West Java. The proportion of active users of LARCs in Pameungpeuk is also considerably low (10.66%) ^15, 16^.

There are many factors that influence the lack of availability and access to LARCs method. These factors include fertility, lack of knowledge and other reasons related to methods that can act as barriers to the use of LARCs ^17^. The factors than can inhibit access to the use of LARCs are higher costs for individuals, lack of trained health providers, short acting methods are widely available in rural areas, long distance to the clinic or hospital and medical barriers ^18, 19^. Some people have negative beliefs and misunderstanding about LARCs, additionally the lack of knowledge and skills of health workers can also influence access to the use of LARCs methods ^20, 21^.

Factors that are suspected to be related to the utilization of LARCs are age, education, family income, cost of contraception, knowledge, beliefs, attitude of the family planning client, exposure to information on LARCs, contraceptive related skills, support from partners, friends, health workers, and also support from leaders in the community. There are, to our knowledge, no studies that have documented the factors associated with the lack of use of LARCs in the Pameungpeuk region. This study therefore aims to assess the factors associated with the utilization of LARC methods among family planning clients in the Pameungpeuk Rural Hospital, West Java, Indonesia.

## Methods

### Study design

This was a hospital-based cross-sectional study. Eligible study participants were women seeking family planning services during the study period from the 15 ^th^ of October, 2015 to the 15 ^th^ of February 2016 at the Pameungpeuk Rural Hospital, which is a health institution that serves over 6000 annual pregnancies in the south of Garut, West Java Province, Indonesia.

### Data collection

An interviewer-administered questionnaire ^22^ was used to collect data for this study. The questionnaire sought information on demographic characteristics, socio-economic status, reproductive history and utilization of modern contraception. Questionnaire items were developed to assess factors associated with the utilization of LARC methods, and included knowledge, beliefs, attitude, exposure to information on LARCs, skills of the health workers, and support from partners, friends, health workers and community leaders. We also sought to foster awareness among midwives and nurses who administer family planning services at the health institution and were asked to administer questionnaires; they were sensitized to the purpose and objectives of the study, and how to administer the questionnaire.

The instrument used in this study was a questionnaire containing questions related to factors associated with the utilization of long-acting reversible contraceptives in the Pameungpeuk Rural Hospital work area. The questionnaire consisted of questions about the behavior of use LARCs methods, knowledge, beliefs, attitudes, exposure to information on LARCs, skills of health workers, support of partner, support of friends, support of health workers, and support of community leaders ^22^.

### Participants

The interviewer-administered questionnaire ^22^ was conducted with consenting consecutive clients as they accessed family planning services until the desired sample size was achieved. The study used a simple random sampling technique to avoid bias. The inclusion and exclusion criteria were as follows: women between 15–49 years who attended the family planning clinic at the Pameungpeuk Rural Hospital from the 15 ^th ^of October, 2015 to the 15 ^th^ of February, 2016. Those who did not give consent, those who could not respond to the interview questionnaire or those who incompletely filled out the questionnaire were excluded. The sample size for the study was calculated using the prevalence of contraceptive use in Pameungpeuk (12% for the rural region) from the Indonesian DHS 2013 report. The formula below was used:

$$n=(Z^{2}pq)/d^{2}$$

Where *n* is the minimum sample size, *Z* is the standard normal deviation, set at 1.96, *p* is the prevalence of contraceptive use in the region (0.12), *q* is 1- *p* (0.88). The degree of accuracy was set at 0.05. The sample size was calculated to be 84 women of reproductive age.

### Statistical analysis

After all the necessary data was collected, the data was coded on pre-arranged coding sheets by the principal investigator. Statistical analyses and data entry were performed using the Statistical Package for the Social Sciences (SPSS), version 20.0. We performed statistical analyses using a chi-square test. Descriptive analysis was used to describe the study sample and the results are presented in tables.

### Ethical consideration

This study was approved by The Ethics Committee of Faculty of Medicine, University of Indonesia under number 245/UN2.F1/ETIK/2015. The research proposal was submitted and reviewed by the Research Committee, whereby permission was granted to conduct the research. A written letter of consent was submitted to the health institution to seek permission to conduct this study, which was also granted. Privacy and confidentiality of the clients’ information was observed through the use of data collection with coded identification numbers. Written informed consent was obtained from all participants prior to participation.

## Results

### Socio-demographic characteristics

A total of 84 family planning clients at the Pameungpeuk rural hospital participated in this study. As shown in Table 1, the majority of participants were more than 30 years old (61.90%). More than half of participants had their first marriage and first childbirth between the ages of 20 and 30 years (55.95% and 63.10%, respectively). Most of the participants had a low level of education, i.e. not beyond graduation from elementary school (32.14%). A majority of participants were Muslim (96.42%). In addition, more than half of participants (69.05%) had a low family income (i.e. a monthly income of less than 200 USD), and they thought that cost of contraceptives in Pameungpeuk was quite expensive (53.58%). Utilization of modern contraceptives at the Pameungpeuk rural hospital was dominated by the use of short-acting contraceptives (Non-LARCs), namely injectable contraceptives (59.52%) and pills (15.47%), while the use of LARCs was just less than 30% [IUD (16.66%) and implant (8.33%)]. ( Table 1)

**Table 1.** Socio-demographic characteristics of family planning clients at the Pameungpeuk Rural Hospital.

Variable	N	(%)
**Age**		
< 20 years old	0	(0)
20–30 years old	32	(38.10)
> 30 years old	52	(61.90)
**Age of first marriage**		
< 20 years old	30	(35.71)
20–30 years old	47	(55.95)
> 30 years old	7	(8.34)
**Age of first childbirth**		
< 20 years old	26	(30.95)
20–30 years old	53	(63.10)
>30 years old	5	(5.95)
**Education**		
No education	10	(11.90)
Graduate of elementary school	27	(32.14)
Graduate of junior high school	23	(27.38)
Graduate of senior high school	20	(23.80)
Graduate of college	4	(4.78)
**Religion**		
Islam	81	(96.42)
Non-Islam	3	(3.57)
**Family Income**		
High income (>200 USD per month)	26	(30.95)
Low income (<200 USD per month )	58	(69.05)
**Cost of Contraception**		
Inexpensive	39	(46.42)
Expensive	45	(53.58)
**Use of Modern contraception**		
**LARCs**		
IUD	14	(16.66)
Implants	7	(8.33)
Sterilization (female)	0	(0)
Sterilization (male)	0	(0)
**Non-LARCs**		
Injection	50	(59.52)
Pill	13	(15.47)
Condom	0	(0)
LAM	0	(0)
**Total**	**84**	**(100)**

LARCs, long-acting reversible contraceptives; LAM, lactational amenorrhea method.

### Factors associated with the utilization of LARCS among family planning clients at the Pameungpeuk Rural Hospital

Factors that are suspected to be related to the utilization of LARCs were age, education, family income, cost of contraception, knowledge, beliefs, attitude of the family planning client, exposure to information about LARCs, contraceptive-related skills and support from partners, friends, health workers and leaders in the community. As shown in Table 2, the results of a bivariate analysis of factors revealed that age, cost of contraception, knowledge, beliefs, skills of health workers, and support from health workers were variables that were significantly associated with the utilization of LARCs. Based on the results of chi-square statistical analyses, of the 13 variables suspected as factors associated with utilization of LARCs, only 6 variables were significantly associated with the utilization of LARCs, and were as follows: age (p=0.024), cost of contraception (p=0.022), knowledge (p=0.042), beliefs (p=0.002), skills of health workers (p=0.008) and support from health workers (p=0.014). The other 7 variables, namely education, family income, attitude, exposure to information about LARCs, support of partners, friends and community leaders, were not significant associated with the utilization of LARCs at the Pameungpeuk Rural Hospital ( Table 2).

**Table 2.** Bivariate analysis of factors associated with the utilization of long-acting reversible contraceptives (LARCs) among family planning clients at the Pameungpeuk Rural Hospital.

Variables	Utilization of contraceptives		Total		p-value
	LARCs	Non-LARCs	N	%	
**Age**					
20–30 years old	15	17	32	(38.09)	0.024
>30 years old	6	46	52	(61.90)	
**Education**					
High education	5	19	24	(28.57)	0.212
Low education	16	44	60	(71.42)	
**Family income**					
High income	7	19	26	(30.95)	0.087
Low income	14	44	58	(69.04)	
**Cost of contraception**					
Inexpensive	9	30	39	(46.42)	0.022
Expensive	12	33	45	(53.57)	
**Knowledge**					
Good	13	23	36	(42.85)	0.042
Bad	8	40	48	(57.14)	
**Beliefs**					
Positive	16	24	40	(47.61)	0.002
Negative	5	39	44	(52.38)	
**Attitude**					
Positive	6	22	28	(33.33)	0.593
Negative	15	41	56	(66.66)	
**Exposure to information on LARCs**					
Exposed	9	34	43	(51.19)	0.378
Non-exposed	12	29	41	(48.80)	
**Skills of health workers**					
Skilled	17	30	47	(55.95)	0.008
Unskilled	4	33	37	(44.04)	
**Support of partner**					
Supported	12	48	60	(71.42)	0.094
Not supported	9	15	24	(28.57)	
**Support of friends**					
Supported	13	45	58	(69.04)	0.414
Not supported	8	18	26	(30.95)	
**Support of health workers**					
Supported	10	48	58	(69.04)	0.014
Not supported	11	15	26	(30.95)	
**Support of community leaders**					
Supported	10	23	33	(39.28)	0.367
Not supported	11	40	51	(60.71)	
**Total**	**21**	**63**	**84**	**(100)**	

Dataset 1. All raw data and demographic information obtained from subject during the present study https://dx.doi.org/10.5256/f1000research.15755.d228026
Copyright: © 2019 Harzif AK et al. 2019 ${data-license-text}

## Discussion

This study reveals a relationship between maternal age and LARC utilization. At <20 years or >30 years, family planning participants generally prefer high-effectiveness contraceptives such as the IUD, pill, or injections. These findings are consistent with previous research conducted by Tunnisa *et al*. and by Soppeng *et al*. in Pangkep, who found a relationship between maternal age and type of contraceptive use ^23, 24^. The results of this study are also consistent with those of Ama *et al*., who conducted a study in Botswana and found that the age of respondents is significantly associated with contraceptive use ^25^.

The results of our data analysis show that there is no relationship between educational level and LARC use among family planning clients. This suggests that a high level of education does not affect the participants’ decision of which type of contraception to use; respondents with both low and high levels of education are aware of the importance and benefits of contraception, and have been likely made aware by health workers or other sources. The results of this study are also consistent with research conducted by Wa Ode in Pasarwajo, Buton, Indonesia, which found no relationship between the level of education and contraceptive use ^26^.

The income of a family is closely related to their attitude towards contraceptive use; a family’s income is one of the factors that influences their acceptance towards, and the decision to use, new contraceptive innovations. Our results showed that respondents who used LARC and non-LARC contraceptive methods were mainly low-income. Among non-LARC users, only a small percentage had high incomes. This indicates that the desire of couples to accept family planning is still high, despite their low income, as in terms of cost, non-LARC contraceptives tend to be cheaper than LARCs. In this study, there was no significant relationship between family income and LARC contraceptive use (p=0.087). It can therefore be assumed that the level of family income does not affect the purchasing power of respondents to buy contraceptives. This research is inconsistent with research conducted by Maiharti *et al*., who found a significant relationship between family income and the use of contraceptive methods ^27^.

Our results show that there was a significant relationship between the cost of contraception and the use of hormonal contraception. Thus it can be said that the costs incurred for contraceptives are related to the choice of which contraception to use; the cost of non-LARCs tends to be less than that of LARCs. This study was consistent with research conducted by Aryanti *et al*., which showed a relationship between contraceptive costs and contraceptive selection ^28^. The results of this study are also in accordance with research conducted by Fienalia *et al*. at the Pancoranmas Health Center, Depok, which found a relationship between the affordability of contraceptive costs and the use of LARCs ^29^. This study was done before the new National Health Insurance Scheme was rolled out and widely available. National health insurance has in recent years, overcome many of the cost barriers to contraceptive use, including use of LARCs. Hartanto *et al*. found that there was a correlation between the use of contraceptive with support from husband or their partner ^30^. Under ideal circumstances, couples should jointly choose the best method of contraception, mutually cooperate in their use, jointly pay for their expenses and take into account any dangerous side-effects. The results of statistical analyses showed no relationship between a partner’s support and the use of LARCs. Therefore, it can be assumed that a partners’ support does not affect the choice of the woman to seek family planning services and to continuously use contraceptives. This study was inconsistent with research conducted by Rizali *et al*., which found a relationship between support from the partner and the use of pill-based contraceptives ^31^. This research is also inconsistent with a study conducted by Setyawati in the Mamojang district of Makassar City, Indonesia, which found that support from the partner will encourage participation in family planning ^32^.

The results of our statistical analysis indicate that there is a relationship between the information provided by health-care personnel and the use of LARCs. This means that family planning clients already have information regarding contraceptive use from other sources. Others sources are from doctors, books, schools, television or friends. Our results showed that family planning LARC users were more likely to have not received information from family planning officers, while non-LARC users were more likely to have received information from family planning officers. This shows the lack of information dissemination from family planning officers to the community, or the lack of active support from health workers at the Pameungpeuk hospital towards the use of LARC methods, with the end result being a lack of information among the public. Based on information from one midwife who served in the family planning service, it is suspected that the health officer in charge is less active in conducting counseling regarding family planning by LARC methods. This research was consistent with Siddik *et al*., which found that there is a relationship between information dissemination and support from the family planning officers with the choice of contraceptive method. This is also consistent with research conducted by Rizali in the Mattoangin district of Makassar City, Indonesia, which showed a relationship between information dissemination by family planning officers and contraceptive use ^31, 33^.

## Conclusions

This study found a significant relationship between LARC use and the age of women (p=0.024), cost of contraception (p=0.022), knowledge (p=0.042), beliefs (p=0.002), skills of health workers (p=0.008) and support from health workers (p=0.014). Education (p=0.212), family income (p=0.087), attitude (p=0.593), exposure to information about LARCs (p=0.378), support from partner (p=0.094), support from friends (p=0.414) and support from community leaders (p=0.367) all had no significant relationship with LARC utilization. Therefore, improving the skills of health workers related to contraception needs to be developed, besides that family planning acceptors are expected to play an active role in any activities related to contraception as well, especially in the use of long-term contraceptive methods at Pameungpeuk rural hospitals. Related sectors should seek to increase community knowledge regarding contraception and clarify of myths regarding the use of long-term contraceptive methods.

 These findings highlight a critical need to improve partners’ understanding of the importance of using LARC methods, to support females in the use of contraception. This also requires increased knowledge dissemination by health-care workers. We recommended that family planning officers improve counseling to mothers and encourage them to remain active contraception users. They must also aid in the community’s understanding of the importance of contraceptive use, especially the utilization of long-term contraceptive methods.

## Data availability

**Dataset 1. All raw data and demographic information obtained from subject during the present study.** DOI: https://doi.org/10.5256/f1000research.15755.d228026 ^34^.

### Extended data

Extended data contains the questionnaire (Bahasa and English versions) and how data were measured: https://doi.org/10.6084/m9.figshare.9734561.v2 ^22^.
