# Supplementary material for: Factors associated with the utilization of long-acting reversible contraceptives among family planning clients at the Pameungpeuk Rural Hospital, Indonesia
Source: F1000Res. 2019 Sep 17;7:1891. Originally published 2018 Dec 4. [Version 2] doi: 10.12688/f1000research.15755.2 (PMC6852292; doi:10.12688/f1000research.15755.2)
Supplement: Peer review history

# Peer review: Reviewer response for version 2

**DOI:** 10.5256/f1000research.22372.r54056

**Published:** 2019-11-12

**Authors:** Douglas Storey

**Affiliations:** 1 Department of Health, Behavior & Society, Johns Hopkins Bloomberg School of Public Health, Baltimore, MD, USA

The authors have addressed my concerns. I have no further comments to make.

I confirm that I have read this submission and believe that I have an appropriate level of expertise to confirm that it is of an acceptable scientific standard.

# Peer review: Reviewer response for version 1

**DOI:** 10.5256/f1000research.17197.r46943

**Published:** 2019-05-23

**Authors:** Douglas Storey

**Affiliations:** 1 Department of Health, Behavior & Society, Johns Hopkins Bloomberg School of Public Health, Baltimore, MD, USA

The manuscript provides a useful, localized analysis of contraceptive method choice in a particular area of West Java, but fails to provide adequate details about how key variables were operationalized, making it difficult to judge the quality of the analysis and some of the conclusions reached. The manuscript could be indexed with major revisions.

 Detailed comments and questions:

 (p1) In the Abstract: “...the use of short-acting contraceptive is not effective enough for use.”

- Contraceptive should be plural (contraceptives).
- “Not effective enough for use” is an overstatement. Injectables and oral pills are highly effective at preventing pregnancy when used properly. They are, however, less efficient than longer acting or permanent methods when used for longer term spacing or limiting of childbirth because it is easy to skip a treatment or take a short break from use for economic or other reasons, which can result in unintended pregnancy. The authors should clarify the distinction between effectiveness of the methods and efficiency or practicality with regard to achieving fertility objectives.

(p3, column 2, 1 ^st^ full paragraph): “Based on the 2015 results...”

- Authors should consider updating these contraceptive use statistics with the more recent results from the 2017 Indonesia Demographic & Health Survey (Survei Demografi dan Kesehatan Indonesia 2017).

(p3, column 2, 2 ^nd^ full paragraph):  “Widespread of myths and misunderstandings...”.

- Delete word “of”
- “Myths” is a poorly defined word that is commonly misused to refer to negative beliefs or attitudes about contraceptive methods. Saying that someone believes in a “myth” suggests that they are superstitious and ignorant. It is more appropriate and more accurate to simply say that some people have “negative beliefs and misunderstandings” about LARCS.

 (p3, column 2, Section on Data Collection):

- My most serious concerns about this manuscript regard the lack of clarity about how some key variables are conceptualized and measured. Authors should provide full descriptions of how variables were operationalized in the interview and/or manipulated (coded or recoded) for analysis. Specifically:
  - Nowhere in the manuscript is there a description of how “knowledge” was conceptualized and measured. Knowledge of what? Of methods available? Of how they work? Of contraindications or side effects? Of how long they can be used? Without this description it is impossible for the reader to know what “Good” or “Bad” knowledge means and therefore to be able to understand how or why his variable might be related to use of LARCs.
  - Similarly, nowhere do the authors describe how “beliefs” and “attitudes” were conceptualized and measured. What beliefs and attitudes were asked about in the interview? Was there a single measure of belief or were multiple beliefs asked about and used to construct a knowledge scale or index? Were respondents asked about the strength of their belief in certain things about FP methods (e.g., “How strongly do you agree with each of the following statements?”) or simply asked, “Do you believe X, yes or no?). What attitudes toward contraceptive methods were assessed in the interview? How were they used to determine if attitudes were positive or negative? The manuscript needs much more detail about how these important independent variables were measured.
  - Similarly, how was “Skills of health workers” conceptualized and measured? What skills were considered? Counseling skills? Clinical skills? How were some workers categorized as Skilled and others as Unskilled? It is impossible to know what this variable means in Table 2.

(p5, 1 ^st^ column):

- Authors find that (perceived) cost of contraception has a significant negative effect on contraceptive use. It may be worthwhile to note that this study was (I believe) done before the new National Health Insurance scheme (Jaminan Kesehatan Nasional/Badan Penyelenggara Jaminan Sosial) was rolled out and widely available. National health insurance has, in recent years, overcome many of the cost barriers to contraceptive use, including use of LARCS.

(p5, Table 2):

- I would find it very helpful to see cell percentages in the Utilization of Contraceptive columns of the table.

(p7, 1 ^st^ column, 2 ^nd^ paragraph): “The results of our statistical analysis indicate that there is a relationship between the information provided by the health-care personnel and the use of LARCs.”

- This comment is related to my concern above about how “health worker skills” was measured. How did the interview determine what information health workers provided to their clients? If the client reported that the health worker did not provide information about LARCS, is that what results in categorization of the health worker as Unskilled? This needs clarification.

I confirm that I have read this submission and believe that I have an appropriate level of expertise to confirm that it is of an acceptable scientific standard, however I have significant reservations, as outlined above.

# Response

**Published:** 2019-05-24

**Authors:** Douglas Storey

**Affiliations:** Johns Hopkins Bloomberg School of Public Health, USA

Dear Authors - When I reviewed your manuscript, I overlooked the fact that the questionnaire was available through a link on the webpage. I can see now how the knowledge, attitudes, beliefs, health worker skills and other independent variables were presented in the questionnaire, but I still don’t know how the multiple measures for knowledge (Q12-Q20) were combined to create “good” vs “bad” knowledge. Same concern for beliefs (Q21-Q26), attitudes (Q17-Q36), exposure (Q37, multiple response) and health worker skills (Q38-Q41). Other independent variables were also constructed from multiple items in the questionnaire. In each case, I recommend that you explain how the analytic variables were created from multiple questionnaire items.

# Response

**Published:** 2019-08-23

**Authors:** Achmad Kemal

**Affiliations:** RSUPN Dr. Cipto Mangunkusumo Hospital, Indonesia

Dear Douglas Storey,

Thank you for the review. We uploaded the revision of our manuscript. The text has been updated to address all of the points. The abstract is updated to clarify the efficiency of short-acting contraceptive. The prevalence of contraceptive methods section has been updated to describe more recent result from the State Ministry for Population and National Family Planning Coordinating Board (BKKBN) in 2017. Data collection section has been update to provide adequate details about how some key variables were conceptualized, measured and operationalized in the interview and/or manipulated (coded or recoded) for analysis. In Table 2, the percentages in the utilization of contraceptive has been added in the columns of table.

We carefully have modified the manuscript according to the your instructions. Thank you for your considerations, we looking forward to any helpful feedback.

Best Regards,

Achmad Kemal

# Peer review: Reviewer response for version 1

**DOI:** 10.5256/f1000research.17197.r41452

**Published:** 2018-12-19

**Authors:** Luis Bahamondes

**Affiliations:** 1 Department of Obstetrics and Gynecology, University of Campinas Medical School, Capinas, Brazil

In my opinion, the manuscript submitted by Harzif and co-workers entitled: Factors associated with the utilization of long-acting reversible contraceptives among family planning clients at the Pameungpeuk Rural Hospital, Indonesia” is important for readers for several reasons.

 Indonesia is one of the countries with a large population, high total fertility rate (TFR) and high maternal morbidity and mortality. Furthermore, although the use of modern contraceptives is increased in the last decades, it is mainly based on the use of short acting reversible contraceptives (SHARC), mainly injectables and pills (almost 75%). It is well established that the use of SHARC methods are associated to early discontinuation and high contraceptive failure when compared to users of long-acting reversible contraceptives (LARCs; intrauterine devices and implants). For this reason the government is encouraging the use of LARC methods.  The authors showed data from Pameungpeuk, an Indonesian region which has the second largest population, with the highest TFR in South-West Java and with low use of LARCs (almost 10%).

 The main findings of the study were that the use of LARCs were significantly associated with age of women, the cost of contraception, knowledge, beliefs, skill of health workers, and support from health workers.

 The main limitation of the study was that it was a cross-section design and the number of interviewed women, which cannot allow to generalized the results to a large country like Indonesia. Additionally, it could encourage others to assess other regions to evaluate also the use of LARCs.

I confirm that I have read this submission and believe that I have an appropriate level of expertise to confirm that it is of an acceptable scientific standard.
